# Technology and older adults in British loneliness policy and political discourse

**DOI:** 10.3389/fdgth.2023.1168413

**Published:** 2023-08-16

**Authors:** Elian Eve Jentoft

**Affiliations:** Faculty of Social Sciences, Oslo Metropolitan University, Oslo, Norway

**Keywords:** loneliness, policy analysis, older adults, technology, WPR, United Kingdom

## Abstract

**Introduction:**

This article provides an analysis of recent loneliness policy and political discourses from the United Kingdom pertaining to older adults. Although government asserts that several groups in society are “at risk” of loneliness, older adults remain the most frequent targets of policy interventions. Technology is positioned as playing a role in the causation and alleviation of loneliness. Little research has examined loneliness in political discourses.

**Methods:**

With a focus on how loneliness intersects with themes of technology and aging, this article presents an analysis of discourses guided by Bacchi's What is the Problem Represented to Be (WPR) framework. It endeavors to answer the following questions: What is the problem of loneliness among older adults represented to be, and what is the role of technology in this context – problem or solution?

**Results:**

In the discourses, assertions are made that issues of loneliness, societal change and digital exclusion are intertwined. Lonely older adults are problematized as hard to find and thus connect with interventions, warranting surveillance measures like loneliness heatmaps. Technological interventions to assist older adults in maintaining independence and connections to social networks are often proposed as solutions. The findings indicate dominant discourses position older adults primarily as subjects in need of care and as non-users of technology. Technology is positioned as a cost-effective tool to fill gaps in an overburdened and under-funded social care system that compounds issues of loneliness.

**Discussion:**

The author argues the neoliberal and stigmatizing undertones within the corpus may undermine efforts to combat loneliness. Further, austerity is silent in the dominant problematizations of loneliness, foreclosing upon alternatives that problematize loneliness as resulting from neoliberal policies that continue to dismantle public infrastructure and social care.

## Introduction

In recent years, several countries have enacted policies on loneliness reduction and prevention (1–4). Among the first and most proactive is the UK, where the devolved governments have developed targeted loneliness strategies and conducted extensive follow up in the years that followed (5). Findings from the present study reveal that in these policies, assertions are often made that issues of loneliness and digital exclusion are intertwined, with technology positioned as playing a role in the causation and alleviation of loneliness. Technologies that assist older adults in maintaining independence and connections to their social networks are frequently included among policy solutions. Previously, loneliness has been primarily approached as a problem for older adults in UK policy (6). Although the authors of most policies assert that various groups in society are “at risk” of loneliness, some continue to focus predominantly on older adults (7–9).

This article presents an analysis of discourses guided by the thinking of Carol Bacchi. Bacchi argues that problems do not exist independent of the policymaking process itself, but are, rather, “constituted” through policymaking (10). Examining solutions proposed within policy, Bacchi argues, can grant insight into implicit political problematizations, of which policymakers themselves may be unaware (11). Bacchi encourages researchers to seek out alternative discourses and consider how other discursive formations could produce very different solutions (and, theoretically, quite different problems). This article examines loneliness policy and political speech as it pertains to older adults and their (non)use of technology. In doing so, it endeavors to answer the following question: What is the problem of loneliness among older adults represented to be, and what is the role of technology in this context–problem or solution?

## Background

The UK is well-known for its work dedicated to loneliness reduction. This is due, in part, to the government's creation of a Minister of Loneliness in 2018, a move which drew international media attention (12). Since the final report of the Jo Cox Commission on Loneliness in 2017, three of the UK's devolved governments (England, Scotland and Wales) and several municipalities have released loneliness-reduction strategies. Most strategies focus on two primary demographics due to their propensity towards higher reported levels of loneliness: the young and the old (13).

Although loneliness is a minority experience among older adults (14, 15), media representations in Western societies often present loneliness as a common and inevitable part of the aging process (16, 17). Research can add to this misconception (14). For example, in 1948, the earliest large-scale study of older people in the UK stated, “A distressing feature of old age is loneliness. All who have done welfare work among the old have found it the most common, if at the same time the most imponderable, of the ills from which the aged suffer, and its frequency was amply confirmed by our study” [Sheldon in (18)]. The NHS has perpetuated this discourse (15). These discourses have impacted policymaking. Thus, loneliness has traditionally been constructed in policy as an issue affecting older adults (6).

Most research on loneliness among older adults has stemmed from the fields of psychology and medicine. It has consequently focused on risk groups and health problems that are claimed to arise due to loneliness (19). Victor, Scambler and Bond argue the pathologizing of loneliness has led to a predominant focus on negative rather than positive sides of aging in research (14). Others have cautioned that pathologizing loneliness can limit what we can conceive of doing about the cultural, social, and structural dimensions at play (20, 21). This focus has had clear influences on policymaking, which increasingly understands loneliness as a public health issue, drawing talking points about the urgency of the situation from epidemiological studies (Sandset, Jentoft and Haldar, in press).

Several reviews of research on loneliness interventions have been performed. Hagan and colleagues found most studies to be of poor quality (22). They found the most successful of the 17 interventions under review to be those including technological interventions (22). A review of reviews found that poor description of the interventions, theoretical frameworks and target groups made comparing studies and drawing conclusions difficult (23). Findings on the impact of social media on loneliness in older adults are mixed (24). Some researchers have argued that the tendency to define loneliness as a problem of deficient social networks individualizes the problem (and interventions), ignoring broader social contexts (14, 20, 25). While psychological interventions have shown promise, Hagan asserts these interventions neglect other issues older people face, such as transitions to care housing, losing a spouse, or declining health (15). He argues, due to the stigma associated with loneliness, interventions that target it directly may be avoided by the target group (15).

Older adults' non-use of various technologies, especially the internet, is often described as a “gray digital divide”. Because of this perceived rift in online engagement and its proposed impact on loneliness, digital skills training is commonly suggested as an intervention for lonely older people (22, 24). A review by Dickinson and Gregor showed several studies had difficulty determining if the communication technologies or face-to-face training component reduced loneliness in older study participants (26). It has also been suggested that young people, assumed to be strong users of technology, should be trained to teach older adults. Researchers have found young people may not always be optimal instructors, as they may explain processes too quickly without providing opportunities for experimentation and repetition (27–29). Studies reveal tech-savvy older adults may represent a better match for instructing older adults (28–30). This may be because, as older adults themselves, they have a shared “lifeworld”, having grown up without these technologies. They are thus able to empathize with and recognize the challenges older adults face when accessing technology (28, 29). Geertz and colleagues suggest a need for a unified approach of help from family paired with professional skills training (28).

Although a lack of access or skills may act as deterrents, some researchers have found beliefs and attitudes about social media to play a role in non-use. These include lack of interest, beliefs that social media provides only superficial connections, disdain for the type of content found on social media and privacy concerns (24, 31). Web design may also not cater to the needs of older adults (31).

Research with avid older users of social media has found that a primary motivation was keeping up with established friends and family (24, 31). Olsson and Viscovi found the small group of adept internet users from their national sample tended to be younger members of older adult cohorts, highly educated, and “economically advantaged” compared to non-users (32). They maintain policymakers must consider how increased digitalization impacts those who do not fall into this “privileged” group (32).

An individual's self-efficacy, or the belief that one can master a skill, has also been found to play a role in tech adoption (30, 32, 33). Some older adults may feel more comfortable using the telephone to mitigate loneliness (34). Technological interventions can also have unintended consequences. These can include bringing limitations due to frailty into the foreground, disappointment when communication is of a lesser quantity or quality than expected, or the inadvertent creation of family tensions (35).

The belief that older adults lag in tech adoption is not necessarily accurate. In some cases, older adults have acted as early adopters (28, 36). However, those who adopt technology are often treated in public discourse as exceptional cases who follow youth trends, rather than as contributors to tech trends in their own right (37). An analysis of the discourses in research on game development for older adults found games created for this audience tend to serve instrumental purposes (for example, maintaining physical coordination, as socialization stimulus or for brain training) rather than existing for the sheer joy of leisure (38).

Attitudes and beliefs among older adults can also impact willingness to accept assistive devices. Some may refuse assistive and care technologies designed for older adults because they are stigmatizing (39–41) or do not meet their actual needs or desires (39–43). Some may refuse such supports if they fear they will reduce the personal help they receive at home. Others may wish to maintain an “undisturbed” environment, or be resistant to change (39). Greenhalgh and colleagues discovered that successful implementations of technology often involved some form of “bricolage”, or creative modification which standardized assistive technologies do not always facilitate. This adaption often requires help from the user's social network, something that some older adults may lack (43).

Across Europe, how aging is problematized has changed dramatically throughout the years (44). Anxieties about increases in the older demographic due to the post-war baby boom and medical/societal advancements, have long been expressed in Western governance, often in negative terms. Frequently envisioned as a “silver wave”, this discourse signifies an approaching tsunami of older people for whom the logistics of care will fail. The underlying assumption is that the dependence of this population on the state will have devastating impacts on society (45, 46). The demographic shift is problematized in the UK against a backdrop of austerity measures which first began over a decade ago. The reforms resulted in dramatic cuts to day programs, libraries, and bus routes integral to the sociality of older people. Christensen and Pilling contend that reductions in social care spending despite projected demographic changes and eligibility restrictions are rarely problematized in UK policymaking (47). Dominant public discourses in the UK hold independence as the moral ideal for a good life in advanced age, whereas dependency is constructed as an “inferior state” (48). Simultaneously, older adults are frequently represented as passive non-actors in public discourse (48, 49).

Concerns about an aging population have fostered an emphasis on active aging as an ideal. Active aging counters negative discourses on aging with (normative) positive portrayals of lifestyles older adults should strive for. The UK policy *Opportunity Age*, which has a heavy focus on active aging, equates the concept with continuing as economic contributors and maintaining activities, even in the face of declining health (50). Part of this discourse includes the concept of “ageing in place”, which encourages adults to live at home as long as possible (41, 45). Similarly, the “aging and innovation” discourse promotes technological solutions to challenges people face in aging (42). When it comes to telehealth and telecare technologies, a tendency among policymakers is to adopt a “modernist” discourse that sees these technologies as a “rational and cost-effective” solution to the impending “problem” of an aging population (41, 46). This discourse simultaneously evokes utopian visions of a future where older adults are empowered citizens, thriving in their smart homes (46). These technologies are desirable because they allow older people to safely age in place without additional strain on the care system. Neven and Peine argue the aging and innovation discourse acts as a device that legitimizes economic investment in technologies in the name of cost-reduction. Simultaneously, it relies upon a negative view of the demographic shift to succeed (42).

There is evidence that negative discourses about the “impending burden” of an aging society are internalized by older adults, resulting in behaviors that counteract efforts to reduce loneliness. Goll and colleagues found some London-dwelling older adults rejected seeking help for loneliness, fearing it would spoil their identity as an independent member of society. Some failed to apply for social programs because help-seeking was seen as amoral and taking advantage of others' kindness (34). Other research has shown that services for older adults are seen as stigmatizing, second rate, and signaling poverty (41). Although their study looked at the uptake of telecare and assistive devices, the beliefs of participants in Greenhalgh et al.'s study were also telling considering issues of loneliness and social isolation. Most participants did not wish to bother their families, and one had completely cut off contact to avoid becoming a burden (43). These findings are understandable considering how British care discourses construct care recipients as a lesser-citizens and burdens on society. Thus, dependency on others becomes a thing to be feared and avoided (48).

Previous discursive research has focused on claims-making activities among charitable organizations, economists, the church (19) and the media (16) about loneliness and older adults in the UK. Little research has examined the discourses in the British political sphere since the shift toward political prioritization of loneliness. Considering the continued discourse about loneliness, the Covid-19 pandemic, and the subsequent government responses, the analysis of the policies presented here is particularly timely.

## Materials and methods

Analysis was conducted using a four-phase procedure inspired by LeGreco and Tracy's discourse tracing (51), wherein the main analysis phase was informed by Bacchi's WPR framework (11, 52). First, research on loneliness in the UK, news media and relevant briefings from interest groups were explored along with documents that would later form the corpus, to acquire an overview of the broader context. While the initial point of departure derived from the English government's loneliness strategy *A Connected Society* (53), this early phase aided the identification of what LeGreco and Tracy call a “turning point” or “rupture” (51), in this case: the Jo Cox Commission report.

The data for this study includes white papers, green papers, internal briefings, parliamentary debate transcripts, press releases, public speeches and statements made by policymakers to the press. Documents were procured through searches for the term “loneliness” in www.gov.uk, www.wired-gov.net and www.hansard.parliament.uk. Additionally, some texts were found via snowballing through their mention in another document within the corpus. Items from the press were obtained via Google News and were included if written by or containing quotes from policymakers. Policymakers include not only political actors who are elected or appointed to office, but also others responsible for policy formation, such as civil servants and advisors.

The broad range of document types was brought in to examine if problematizations shift in different contexts. Additionally, the discourses included in formal policy documents are not necessarily representative of the range of problematizations emphasized by other political actors, for example, by members of parliament who are not part of the current administration. These problematizations may be silent in policy but have the potential to be captured in debate transcripts or interviews with the press. Press releases reveal how loneliness is problematized through implementation in government initiatives and programs. This is important, given Bacchi's emphasis on beginning with the solutions themselves (see below). A selection of documents with similar themes, but which did not concern loneliness (for example, digital strategies) were also analyzed for the sake of discovering alternate problematizations and challenging assumptions.

The corpus was organized and analyzed chronologically. Chronological ordering facilitates the location, not only of discursive shifts that occur over time, but also contextual nuances, tensions, discursive alternatives and silences (51). Preliminary reads of the ordered documents were performed, in which they were subjected to sorted grading and categorized by document type and according to specific areas of interest. During this phase, notes were taken on potential patterns and themes to pursue. For the purposes of this paper, documents lacking mention of older adults were extracted from the corpus. Because the area of interest concerns how loneliness and technology intersect, documents without mention of technology were also removed from the dataset.

In total, 100 documents of various lengths were analyzed for this paper, including 13 loneliness strategies from different levels of government. The UK represents a unique case. As previously mentioned, it does not have one overarching strategy. Instead, each devolved government (excluding Northern Ireland) has developed its own. Several local strategies down to the council level also exist. These can offer glimpses into how, for example, boroughs with different demographics problematize loneliness within their localities. The documents included span from 2017 to early 2021. The rationale for beginning with 2017 is that The Jo Cox Commission delivered its seminal report that year (54). This report is widely recognized as influencing the appointment of a Minister for Loneliness and forms the foundation for much of the policy that would follow (55). Continuing data collection through the beginning of 2021 provided insights into how discourses on loneliness' relationship to technology may have been impacted by the Covid-19 pandemic. Ending data collection in early 2021 prevented the corpus from becoming too large to analyze thoroughly, given the increased attention on loneliness at the time. For an overview of the number and type of documents included, see Tables 1, 2.

**Table 1:** Corpus document overview.

Document type	#
Loneliness strategies	13
Parliamentary debates	11
Briefings/reports	26
Public inquiries	4
Political speeches	1
Written question	5
Government press releases	18
Press/blog statements	7
Other	5
Non-loneliness documents	10
Total	100

**Table 2:** Documents included in the corpus.

Document	Type	Publishing organisation	Year
Loneliness and local communities	Debate Transcript	Hansard House of Commons	2017
Effect of loneliness on local communities	Briefing/Report	Commons Library	2017
Tackling loneliness with technology	Blog/Press	TheMJ.com/Graham Allen (Hampshire County Council) & David Rees	2017
Combatting loneliness one conversation at a time	Briefing/Report	Jo Cox Commission on Loneliness	2017
Loneliness and social isolation in the London borough of Hounslow	Strategy	London Borough of Hounslow	2017
Tackling loneliness in Merton	Strategy	Merton Council Task Group	2017
Inquiry into loneliness and isolation.	Inquiry	National Assembly for Wales	2017
Combating loneliness in Southampton	Inquiry	Southampton City Council	2017
Loneliness decision Appendix 1	Other	Southampton City Council	2017
Community life survey: focus on loneliness 2017–18	Briefing/Report	Department for Digital, Culture, Media & Sport	2018
Government uses innovative tech companies to tackle rural isolation and loneliness	Press Release	Cabinet Office, CBE MP	2018
Tracey crouch speech at public health England annual conference	Speech Transcript	Department for Digital, Culture, Media & Sport and Crouch, T MP	2018
A connected society	Strategy	Department for Digital, Culture, Media & Sport	2018
Loneliness. Volume 648: debated on Thursday 1 November 2018.	Debate Transcript	Hansard House of Commons	2018
Loneliness strategy. Volume 647: debated on Monday 15 October 2018.	Debate Transcript	Hansard House of Commons	2018
ExtraCare: building better lives for older people	Press Release	Innovate UK & UK Research and Innovation	2018
Behind Britain’s loneliness problem	Blog/Press	Natasha Hinde/Huffington Post UK	2018
Combating loneliness—a guide for local authorities	Briefing/Report	Local Government Association, Campaign to End Loneliness & Age UK	2018
Loneliness—How do you know your council is actively tackling loneliness?	Briefing/Report	Local Government Association	2018
Loneliness- What characteristics and circumstances are associated with feeling lonely?	Briefing/Report	Office for National Statistics	2018
PM launches Government’s first loneliness strategy	Press Release	Prime Minister’s Office, Civil Society & Theresa May MP	2018
In Awful Isolation	Blog/Press	Rachel Reeves/Fabian Society	2018
A connected Scotland	Strategy	Scottish Government	2018
Social isolation and loneliness strategy 2018–2027	Strategy	South Ayrshire Council & NHS Ayrshire & Arran	2018
Why tackling loneliness needs to be an intergenerational game	Blog/Press	Ummuna, C./Huffington Post UK	2018
Welsh government consultation document—connected communities	Inquiry	Welsh Government	2018
An overview of reviews: the effectiveness of interventions to address loneliness at all stages of the life-course	Briefing/Report	What Works Wellbeing, HM Government	2018
Loneliness—question for department for digital, culture, media and sport. U│N 1,28,492, tabled on 20 February 2018	Written Question	UK Parliament	2018
Tackling loneliness	Briefing/Report	House of Commons Library	2019
Loneliness annual report—the first year	Briefing/Report	Department for Digital, Culture, Media & Sports, Office for Civil Society	2019
New fund for frontline organisations tackling loneliness.	Press Release	Department for Digital, Culture, Media & Sports, Office for Civil Society	2019
“Smart homes” to help older and disabled people get digital skills and tackle loneliness in rural areas	Press Release	Department for Digital, Culture, Media & Sports, Office for Civil Society	2019
Free TV licences: over-75s. Volume 661: debated on Tuesday 11 June 2019.	Debate Transcript	Hansard House of Commons	2019
Older persons: provision of public services. Volume 798: debated on Thursday 13 June 2019.	Debate Transcript	Hansard House of Lords	2019
Reaching out. Guide to helping principal and local councils tackle loneliness.	Briefing/Report	Local Government Association & National Association of Local Councils.	2019
Wealden District Council: tackling loneliness. Public Health Case Study.	Briefing/Report	Local Government Association	2019
Digital inclusion guide for health and social care.	Briefing/Report	NHS Digital	2019
Social isolation and loneliness needs assessment and strategy	Strategy	North Somerset Council	2019
Weldmar hospice care: digital inclusion project	Blog/Press	The DCMS Digital Skills Partnership Blog	2019
Welsh government consultation—summary of responses.	Inquiry	Welsh Government	2019
Transporting people to creativity in Boston and South Holland	Blog/Press	Arts Council England	2020
Government’s work tackling loneliness	Other	Department for Digital, Culture, Media & Sport & Office for Civil Society	2020
Grantee list—loneliness interventions	Other	Department for Digital, Culture, Media & Sport	2020
Loneliness Annual Report January 2020	Briefing/Report	Department for Digital, Culture, Media & Sport & Office for Civil Society	2020
Wellbeing and loneliness—community life survey 2019/20	Briefing/Report	Department for Digital, Culture, Media & Sport	2020
Social cohesion—community life COVID-19 Re-contact survey 2020	Briefing/Report	Department for Digital, Culture, Media & Sport	2020
Wellbeing and loneliness—community life re-contact survey 2020	Briefing/Report	Department for Digital, Culture, Media & Sport	2020
Government launches plan to tackle loneliness during coronavirus lockdown	Press Release	Department for Digital, Culture, Media & Sport & Office for Civil Society	2020
Loneliness minister: write letters to people isolating at home	Press Release	Department for Digital, Culture, Media & Sport	2020
Community groups tackling loneliness to benefit from £4 m fund	Press Release	Department for Digital, Culture, Media & Sport	2020
Government announces £7.5 million funding to tackle loneliness during winter	Press Release	Department for Digital, Culture, Media & Sport	2020
Evidence scope: loneliness and social work	Briefing/Report	Department of Health & Social Care	2020
New technology challenge to support people who are isolating	Press Release	Department of Health & Social Care	2020
Digital innovations tested to support vulnerable people during COVID-19 outbreak.	Press Release	Department of Health & Social Care	2020
Staying mentally well this winter	Other	Department of Health & Social Care	2020
£5 million for social prescribing to tackle the impact of COVID-19.	Press Release	Department of Health & Social Care	2020
Loneliness: older people. question for department of health and social care. U│N 2337 tabled on 15 January 2020.	Written Question	Hansard Library	2020
Question for department for digital culture, media and sport. U│N 47500 tabled on 15 May 2020	Written Question	Hansard Library	2020
Loneliness: coronavirus. question for department for digital, culture, media and sport U│N 81965, tabled on 28 August 2020.	Written Question	Hansard Library	2020
Loneliness: coronavirus. question for department for digital, culture, media and U│N HL8231, tabled on 17 September 2020.	Written Question	Hansard Library	2020
Free TV licences. Volume 669: debated on Thursday 16 January 2020.	Debate Transcript	Hansard House of Commons	2020
Tackling loneliness. Oral answers to questions. Volume 676: debated on Thursday 4 June 2020.	Debate Transcript	Hansard House of Commons	2020
Loneliness: Winter 2020–21. Volume 683: debated on Thursday 5 November 2020.	Debate Transcript	Hansard House of Commons	
Loneliness, social isolation and COVID-19	Briefing/Report	Local Government Association & Association of Directors of Public Health	2020
Jenrick calls for community togetherness to combat loneliness	Press Release	Ministry of Housing, Communities & Local Government	2020
Social capital in the UK: 2020	Briefing/Report	Office for National Statistics	2020
Coronavirus and loneliness, Great Britain: 3 April to 3 May 2020.	Briefing/Report	Office for National Statistics	2020
Rotherham Loneliness Action Plan 2020–2022	Strategy	Rotherham Council Health and Wellbeing Board	2020
Tackling social isolation and loneliness	Strategy	Scottish Government	2020
Be social, be well	Strategy	Loneliness Campaign North Yorkshire	2020
Connected communities	Strategy	Welsh Government	2020
10 tips to help your project reduce loneliness	Other	What Works Wellness Centre, HM Government, Coop Foundation & Community Fund	2020
Disability charities benefit from £2.4 million fund	Press Release	Department of Health & Social Care	2021
Tackling loneliness	Briefing/Report	House of Commons Library	2021
A connected recovery	Briefing/Report	British Red Cross & All Party Group on Loneliness	2021
A connected society? Assessing progress in tackling loneliness.	Briefing/Report	British Red Cross & Loneliness Action Group	2021
Loneliness minister: “It’s more important than ever to take action”	Press Release	Department for Digital, Culture, Media & Sport	2021
Loneliness annual report January 2021	Briefing/Report	Department for Digital, Culture, Media & Sport	2021
Emerging together: the tackling loneliness network action plan.	Strategy	Department for Digital, Culture, Media & Sport	2021
Covid-19 and loneliness. Volume 697: debated on Tuesday 15 June 2021.	Debate Transcript	Hansard House of Commons	2021
Inclusive society. Volume 811: debated on Wednesday 14 April 2021.	Debate Transcript	Hansard House of Lords	2021
Tackling intergenerational unfairness (select committee report). Volume 809: debated on Monday 25 January 2021.	Debate Transcript	Hansard House of Lords	2021
Loneliness: a reading list	Briefing/Report	Hansard Commons Library	2021
Tackling loneliness commons library number CBP8514	Briefing/Report	Hansard Commons Library	2021
How Yorkshire can use satellite technology to tackle loneliness—Amanda Solloway, minister for science, research and innovation.	Blog/Press	Yorkshire Post	2021
£3.4 million National Lottery funding to improve wellbeing in communities across Wales.	Press Release	The National Lottery Community Fund	2021li
Satellite-powered app to spot loneliness in hotspots in UK cities.	Press Release	UK Space Agency	2021
Tackling social isolation and loneliness. A strategy for Hammersmith and Fulham	Strategy	Hammersmith and Fulham Cabinet Member Board for Social Inclusion	2021
Tackling loneliness in Leicestershire	Strategy	Leicestershire County Council	n.d.
Non-loneliness related documents			
Document	Type	Publishing organisation	Year
UK digital strategy 2017	White Paper	Department for Digital, Culture, Media and Sport	2017
Civil society strategy	White Paper	Cabinet Office	2018
The digital age: new approaches to supporting people in later life get online.	Briefing/Report	Centre for Ageing Better	2018
Age friendly and inclusive volunteering review	Briefing/Report	Centre for Ageing Better	2018
How has COVID-19 changed the landscape of digital inclusion?	Briefing/Report	Centre for Ageing Better	2020
Councils awarded £800,000 to build on digital advances made during pandemic.	Press Release	Ministry of Housing, Communities & Local Government	2020
Care robots could revolutionise UK care system and provide staff extra support	Press Release	Department for Business, Energy & Industrial Strategy, Research and Innovation	2019
Connecting Scotland. Investment in digital inclusion exceeds £48 million	Press Release	Scottish Government	2021
Beyond digital: Planning for a hybrid world. 1st report of session 2019–21 HL Paper 263.	Briefing/Report	House of Lords Select Committee on COVID-19	2021
People at the heart of care	White Paper	Department of Health & Social Care	2021

**Figure 1:**
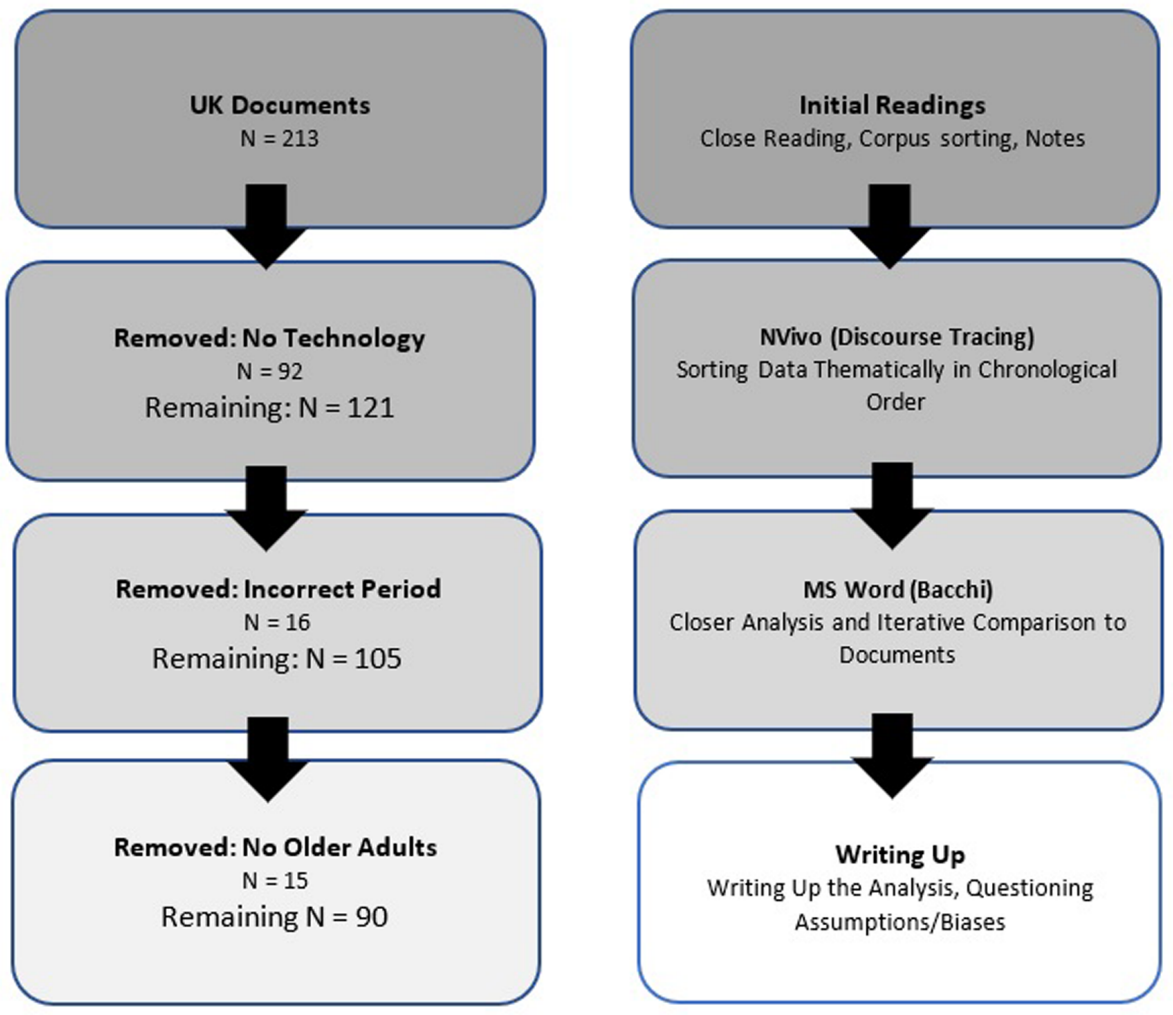
Corpus processing (left) and analysis workflow (right).

The documents were next imported into NVivo where initial themes developed in the first readings were pursued, resulting in the creation of subthemes wherein potential patterns were noted. The phase of discourse tracing which calls for asking explicit questions of the data was informed by the WPR framework (11, 52). For this phase, data excerpts were extracted and collected in Microsoft Word documents according to themes of interest for closer analysis to examine problem representations more closely. The original material was continuously consulted to ensure the validity of the analysis in relation to the larger context. Figure 1 illustrates the document-sorting and analysis processes.

In line with Bacchi's thinking, the approach used here draws upon Foucault, who sees discourse in terms of power. According to Foucault, discourse establishes what can be said or even thought about and who can legitimately speak (56). Similarly, Bacchi argues that policy does not represent problems as they exist out in the world, but instead partakes in their discursive creation, shaping realities (10). These problems are framed in ways that affect how we think about them, and what can be done about them (57). According to Bacchi, “the aim is to understand policy better than policy makers by probing the unexamined assumptions and deep-seated conceptual logics within implicit problem representations” (11). Thus, rather than starting with the problematizations, Bacchi proposes *solutions* provide true insight into what one believes needs to be changed (11). This form of analysis can be a powerful tool to help researchers uncover implicit problematizations, foster sensitivity to silences and an openness to the discovery of alternative approaches to the “problem”.

In accordance with Bacchi's guidance, the analysis centers on *technology*, a common solution proposed to fight loneliness. It then works backwards to understand what these solutions say about what the problem of loneliness among older adults is represented to be. Like other objects, technologies “do not exist as essences; they emerge as ‘objects for thought’ in practices” (52), and thus, not only is loneliness constituted in policy but technologies themselves. “Refracting” the issue of loneliness through technology is a productive way of sifting through norms and values, revealing a spectrum of implicit problematizations (58). By beginning with the proposed “solution”, in this case technology, it becomes easier to uncover how loneliness is produced in these policies as a particular kind of “problem” (10).

Throughout the analysis, careful attention was paid to problem representations across the different levels of discourse, as well as local and political contexts represented in the corpus. The corpus' diversity fostered a deeper understanding of the dominant problem representations contained therein, while also drawing attention to discursive alternatives that arise within various political contexts but fail to gain substantial traction. These may indeed provide opportunities for discovery of other potential problem representations and draw attention to silences and assumptions that lie elsewhere in the corpus.

Although this article focuses solely on the data from the UK, the research reported on in this paper is part of a larger comparative study analyzing policy from Norway and the UK. This backdrop garnered insights into how discourses differ across national contexts and informed the analysis by denaturalizing dominant problematizations. The author additionally conferred with colleagues familiar with loneliness policy to challenge any potential assumptions along with the analysis and findings.

## Results

When exploring the intersection of loneliness, technology, and advanced aging, four problematizations become apparent. All result in the same solution: more technology.

Problem representation 1: Loneliness among older adults is tied to non-use, and thus, exclusion from an increasingly digital society.

Problem representation 2: Loneliness arises from lacking social provisions and infrastructure, such as an overburdened social care and health system.

Problem representation 3: Loneliness stems from geographic spread as a factor of societal change.

Problem representation 4: Lonely elders are difficult to find and assist in managing their loneliness.

In the following, I present these problem representations one by one.

### Problem representation 1: loneliness as digital exclusion and non-use

In the UK, loneliness for some groups is explicitly tied to one's ability to access and utilize technology. Terms like “digital exclusion” and “digital vulnerability” are used to express concerns that increased digitization results in shutting out older people from society. Digital exclusion is thus deemed a major contributor to loneliness, and loneliness is understood as an unintended consequence of modernity. Consider this quote from the Scottish Government's loneliness strategy:

Globally, we are more connected than ever before—with greater access to information and technological resources that enable us to keep in touch across time zones and continents. Yet, despite the prospects it holds for social progress, technology has also presented significant challenges. Those who do not utilise technology or feel less comfortable doing so can be left feeling excluded in a world where online communication and digital services are quickly becoming the norm. However, many people increasingly feel that digital convenience has overtaken face-to-face contact, and technology can actually become a factor in increasing isolation (59).

While highlighting several benefits granted by an increasingly digitized society, online life is conceptualized as replacing face-to-face encounters. This development gives rise to an imaginary in which non-users of the internet become victims, unable to access basic services.

Digital exclusion is additionally tied to loneliness because, as is often argued in the corpus, meeting a worker at the store or the post office may be the only face-to-face contact older people experience throughout the day. Take another example from a House of Commons briefing in a section about social infrastructure and loneliness: “[in] an increasingly individualistic, online based society, coupled with the subsequent closure of physical local services such as post offices; older people may find themselves shut out by the digitisation process” (60). While physical locations are increasingly closed in favor of digital services that are more convenient to the public and cost-effective for government, these developments are said to have had unintended consequences of inconveniencing and excluding those who cannot utilize these new forms of service delivery, namely older people.

In a 2017 Parliamentary debate, former Liberal Democrats leader Jo Swinson told of her grandfather's loneliness following the passing of her grandmother, noting, “Particularly as part of a generation that was not able to embrace the internet, that became a massive isolating factor and another layer of difficulty.” (61). This statement exemplifies an underlying assumption present in the previous excerpts in clearer terms: that by default, members of her mother's generation are assumed unable to access the internet and to have fallen victim to society's ever-increasing reliance upon it.

The solution to this problem then becomes digital skills training. Who policymakers believe should deliver this digital skills training is also explicitly stated. Reliance on family for simple online tasks is often problematized as a negative. According to most documents examined in this study; digital skills training should ideally be provided by young volunteers. This is also envisioned as solving a much problematized lack of inter-generational interaction.

Non-use among older adults is additionally problematized as an issue related to fear and self-efficacy. Particularly prominent is the fear of falling victim to online scams as a deterrence to online engagement. In the English strategy, fraud prevention is reconceptualized as a loneliness intervention:

With the support of the Home Office, the National Trading Standards Scam Marshal scheme will be expanded to improve the resilience of lonely or socially isolated older adults to fraud, scams and financial abuse. We know that those who are isolated may be more likely to be victims of fraud, and likewise, being a victim of fraud itself can be an isolating experience (53).

Eliminating online scams thus becomes a problem of loneliness and reporting scams becomes an act of care toward older people who may fall prey. Similarly, in the Welsh loneliness strategy consultation response document, we read:

[…] it also needs to be recognised that not everyone has the capability or wish to learn how to use digital technology. There also needs to be better awareness of scams and robust and easy to use systems of identifying and reporting scams (62).

Another interesting and somewhat rare admission in this excerpt is the acknowledgement that some may choose to remain offline. Here, the statement is neutral, whereas elsewhere it becomes a behavior to correct, such as in the Southampton Loneliness Inquiry:

[A] Key principle behind the Council's approach –[is] To make sure that we are meeting the needs of the people who can't get online while continuing to work to change the behaviour of the people who choose not to transact with us online”(63).

A core belief behind most documents in the corpus is that non-use is a problem to be corrected for the good of the individual and society. This is in part because of the avenues it opens for cost-effective care and prevention of health concerns associated with loneliness. Cost-effective care via technology would not be as much of a concern, were it not for problematization number two, which follows.

### Problem representation 2: inadequate social infrastructure

In the second problematization, inadequate social infrastructure contributes to loneliness in older adults. These infrastructure issues cover a broad range of services, from the closure of day centers and libraries to the reduction in bus routes and accessible transportation, to the closure of public toilets and inadequate or dangerous sidewalks that prevent frail older adults from leaving their homes. In addition to infrastructure solutions including the reestablishment of day centers and improving public transportation, technology is also recommended^1^. In the context of loneliness policy, technology serves a ternary purpose. It reduces expenditures incurred by an aging society, provides social stimulus, and fills gaps in care provision left by a means-tested social care system described as poorly suited to meet the needs of the public in the following segments.

That people are living longer, and spending more of those years in ill health, is a part of this problematization. In a House of Lords debate, Labour party member Baroness Ramsay of Cartvale says:

[…] social care for our elderly and needy is dismal. Social care provisions are, at best, perfunctory and, at worst, non-existent or unacceptable. The blight of loneliness is increasing and deadly, making long life a misery instead of a blessing (64).

The state of social care in the UK is envisioned as creating a dire scenario in which peoples' lives are diminished to the point that extra years become a curse rather than a boon.

Austerity is ever-present, yet rarely directly mentioned in policy on loneliness. It appears via references to deficits in the availability of social care services and cuts to public sector funding, including that allocated to environmental infrastructure, transportation, libraries, adult day centers and community centers. Although some exceptions exist (9, 65), seldom are cuts directly attributed to the continued impact of austerity measures in the loneliness strategies. The situation is most frequently presented as a regrettable state of affairs, rather than the result of previous policy decisions. Direct and often scathing critiques of social care funding cuts are almost exclusively provided by members of the Labour party in this corpus, mostly in debate transcripts. Conservatives tend to defend these cuts as unfortunate, but inevitable, having had unforeseen consequences in terms of loneliness.

In one example from a House of Commons debate, Labour MP Steve Reed notes that although funding for civil society projects is proposed in response to loneliness, funding cuts enacted since 2010 have reduced the capacity of charitable organizations to act. Even with the proposed funding for public initiatives, he argues that funding for loneliness initiatives is insignificant considering the number of community centers and libraries recently shuttered:

That £1.8 million [in loneliness grants] would reopen just four of the 1,000 children's centres, or nine of the 428 day centres, that have closed under this Government. Unless the Chancellor reverses cuts in public health funding in the Budget, the flourishing of social prescribing and community projects that the Minister wants to see will never happen (66).

Cuts to important public and charitable programs continue, he points out. He argues that the loneliness grants will have little impact unless budgetary decisions are reversed, especially considering the loss of EU funding due to Brexit. Then Loneliness Minister and Conservative party member Tracey Crouch responds, acknowledging that “difficult decisions were taken during difficult times to try to regain an economic balance but those decisions may have had an inadvertent impact on loneliness.” She adds that government going forward will take loneliness into account as policies are made “responsibly” (66).

In addition, statements made by charitable organizations in the Welsh Government's loneliness inquiry argue unstable funding schemes via grants do not consider the time required to train volunteers and causes organizations to regularly lose skilled staff once funding ends (8). In the Welsh Government's loneliness consultation summary, published two years later, some organizations highlight that precarious funding schemes make the continuance of successful interventions tenuous (67).

Technology is recognized in some documents as a method of cutting costs further. Take this example from the Local Government Association's *Combatting Loneliness* guide for local authorities:

Technology can also offer a cost-effective way of providing wider services and support. Technology-based provision may sometimes represent the “best case scenario” in a time of limited resources, even though face-to-face provision may be preferred. The early use of telecare solutions also support independent living, allowing users to remain in their home and community environment for longer, avoiding relocation induced loneliness (68).

That technology is envisioned as supplementing or replacing diminished care systems, offering social engagement (via social robots and telepresence technologies) and access to the outside world when one can no longer access it directly, might be expected. However, in addition to communication technologies, assistive technologies are promoted as remedies for loneliness to the degree that they allow the recipient to maintain a social life and independence.

Everything from care robots to telehealth to automatic door locks to surveillance via sensors and GPS plays a role in loneliness reduction policy by allowing older adults to live self-sufficiently, at home, independent of more costly social care. Technology is also presented to the public in the Welsh strategy as a way responsible older citizens can exercise their independence, defeating loneliness simultaneously as they “[…] use digital technology in a way suited to their personal lives, including management of health conditions, reducing loneliness and social isolation and enjoying the wider opportunities digital can offer” (62).

One interesting example is found in a 2019 scheme in rural West Essex to teach digital skills and “tackle loneliness” among rural-dwelling older people. “The homes […] will see homeowners become trained “digital boomers” to help others improve their digital skills. They will receive a digital assessment, before having their homes ‘kitted out’ in tech.” Recipients are then expected to demonstrate how smart homes enable independence, setting an example for their peers. A subset of younger “digital buddies” is on hand to teach digital skills (69).

Evidence suggests television is a technology used by some older adults to mitigate loneliness (34). Nevertheless, television is not commonly considered preventative against loneliness, at least not within the strategy documents. Perhaps this is because it is considered a passive activity, and therefore in conflict with an active aging ideal. In parliamentary debates, however, parliament members strongly criticized the BBC's decision to end the universal provision of free TV licenses to older adults over age 75, urging the government to step in to reduce loneliness. Conservative House of Lords representative Baroness Redfern calls attention to the issue in a unique way that demonstrates exactly how this problem relates to that of inadequate infrastructure, low incomes, and digital exclusion:

[…] many older people have struggled throughout their working lives to save a little extra for their retirement, but that small pot of savings for a rainy day means that they will not qualify for means-tested benefits. […] These people are likely to have lower disposable incomes after meeting essential disability-related costs, including paying for care and support, so they rely much more on their TV for companionship, entertainment and keeping up to date with news. More generally, we underestimate how many elderly people living on their own rely on their TV to keep them company; as they age, they find human company harder to come by and many do not have access to the internet (64).

While one can imagine this scenario resulting from Covid-19 lockdowns, this excerpt predates the pandemic. This indicates it may also be resultant of social service cuts and strict means-tested care requirements, for, without the lack of social provisions, older adults would not be as distinctly reliant upon the television as their main form of company. Additionally, in both this debate and a similar one in the House of Commons (70), the underclaiming of the pension credit arises. This failure to claim benefits means eligible older adults cannot claim other benefits like the free TV license and bus fares.

The problem obtains a new layer when one examines where blame for the BBC's move toward means-tested free licenses is placed. Conservative party members regard the matter as out of their hands, understanding it as a matter of betrayal by the BBC, which reneged on its promise to maintain the program after its administration was transferred to them (64, 70, 71). Labour party members criticize the Conservative government's decision to relinquish government control of the program, leaving it instead to the BBC's discretion (64, 70). In a House of Commons debate on the issue, then Deputy Leader of the Labour Party Tom Watson chastises the Conservatives, stating, “Public broadcasters should never be responsible for social policy” (70). For Conservative members, the problem is understood as of one of promises broken by the BBC, whereas Labour understands the problem as another dismantled government social program.

Problematization number two is related to problematization number one because if older adults cannot or will not utilize technology, digital services cannot be harnessed. It is further related to the following problematization number three, which sees geographical spread and changes to familial structures as having impacts on loneliness. Technology then offers a means of social contact with distant family members and provides care when family members cannot be carers.

### Problem representation 3: societal change and geographic spread

A related problematization references still another feature of societal change: that families in the UK now live more geographically separated and multi-generational housing is no longer common. Conservative party member Lord Haselhurst explains in a House of Lords debate, “[…] the intergenerational family structure has been weakened, inevitably leaving more for the state to do” (64). However, unlike arguments seen elsewhere, Lord Haselhurst reminds his peers that policymaking decisions and the rising cost of living have forced some young people to live far from their parents, creating this problem. This makes them less available as carers as their parents age. They also enjoy less social contact with their families. This is especially said to be true if the older parent lacks digital skills or access to the internet. The concern is shared across parties.

Intergenerational and co-housing schemes are therefore presented as loneliness interventions that additionally solve the problem of affordable housing shortages. This is also seen as mitigating the costs of an aging society, as indicated by Co-Chair of the Jo Cox Commission on Loneliness, Rachel Reeves, in a 2017 House of Commons Debate. “By bringing younger and older generations together in one household, we potentially address not just the problem of loneliness, but some of the questions about the costs of an ageing society, because we would have younger people looking after older people”(61).

Another example of how societal change is problematized in relation to loneliness can be found in this excerpt from the Welsh loneliness strategy's ministerial foreword, written by Deputy Minister for Health and Social Services, Julie Morgan:

Our modern way of life often serves to isolate us from others. Many of us live alone, work at home, and shop and socialise online. Or we commute long distances back and forth to work and spend long hours working instead of spending time with our families and friends. […] More of us live alone as our population is ageing and we are having fewer children. […] Because of moving for careers or education, more of us live further from our families and the communities we grew up in (62).

This example emphasizes how digitalization can be isolating, along with the need to relocate to seek work or study. However, she adds the relevance of long commutes and working hours as eliminating time that could be used on the social, which one could assume includes socializing with older family members. That recent generations have had fewer (or no) children, resulting in a smaller social network, is also seldom problematized in this way. She takes this one step further in the following paragraph, describing how modern norms contribute:

Shifts in attitude also play a part. Today socialising and investing time in social ties are generally seen as less important than “productive” activities like work and we therefore neglect what appear to be “unnecessary” relationships (62).

Like the above, this suggests that loneliness is a problem of prioritizing economic opportunities over relationships. For older retirees who stand outside of working life, the lesser availability of younger family and friends due to these social and economic changes may be isolating.

In this problem representation, solutions such as those offered for the previous one also become lucrative compared to the higher costs of expanding social care programs. E-health and digital care solve the problem of fewer family members living in the vicinity who can provide direct care. Digital contact with relatives is an approach to the issue that remedies the lack of direct contact. The English strategy, *A Connected Society* states:

And, in the 21st century, digital infrastructure is also a key tool to bring us together, even when we can't be physically in the same place. This can be especially important to people with mobility problems, or families and friends separated by distance (53).

This quote highlights how digital infrastructure enables not only those living at a distance to keep in touch, but also benefits those who are unable to enjoy face-to-face contact because of illness or disability.

The North Somerset loneliness strategy states how becoming a digital elder is considered important to families separated by distances:

“Using IT to keep in touch with known family and friends spread geographically, making regular visits difficult, is favoured where IT use is accessible. […S]ome older people report that the IT training course itself, enabling them to do this was more enjoyable, as [it] was seen as a social face-to-face event in itself. […]” (72).

This anecdotal account brings to mind findings from studies of digital skills courses and loneliness that found it hard to determine whether the face-to-face contact while learning, or the internet use reduces loneliness (26).

### Problem representation 4: lonely people are hard to find

In a 2019 House of Lords debate, Lord Foulkes stated, “Loneliness may be out of sight and out of mind, almost by definition […]” (64). Indeed, one loneliness problematization found across documents is the challenge of identifying lonely people in need of interventions. This is said to result from several issues, ranging from the stigma of loneliness, to the inability of some frail older adults to leave their home. The Merton City Council strategy focuses on:

[…] ways to identify “hidden citizens”; those who are lonely but not connected or known to services in their community. Loneliness is a personal and stigmatised experience therefore it can be difficult to identify people suffering from it (7).

The strategy later states that some will be too embarrassed or lack the confidence to reach out to organizations. This problematization also ties back to the problem of inadequate social care, as they do not come into contact with workers who might identify ways to help them meet their social needs (7). As mentioned in problematization two, additional barriers include poor public transport, degraded local infrastructure that prevents leaving the home and poverty.

Speaking of a survey by Gransnet, a British social media platform for older adults, former Co-Chair of the Jo Cox Commission on Loneliness, Rachel Reeves, states in a House of Commons debate, “[…] the vast majority of people would rather share their feelings of loneliness online than with their friends and family. That might be the quintessentially British thing to do, but it also means that too many people suffer in silence” (61). Loneliness is said to be so stigmatized that some may rather turn to technology than their families. She additionally calls attention to a troubling feature of British social norms: keeping a stiff upper lip, no matter the cost.

The National Assembly for Wales' inquiry describes a “cycle of loneliness” in which “… people are embarrassed to admit they need help more generally and withdraw from society. The more disengaged they become, the more likely they are to become lonely and isolated and less likely to access the help they need” (8). This presents another reason people may not be known by social care agencies: they are ashamed of needing any help at all, and therefore do not receive help to access the community.

Another aspect of the discourse concerns how information about loneliness interventions is dispersed, often via digital portals. As the Hounslow loneliness report suggests, residents may also be unlikely to self-refer due to stigma (9). While these portals are praised, it is acknowledged that some lonely older people will remain unable to access information about these services:

… even if more effectively developed, such online portals are unlikely to offer a realistic route to support for most older people experiencing loneliness and isolation. Therefore services which can provide in-person support to help lonely people identify appropriate ways in which their needs for connection can be met will remain vital (9).

In this way, the problem of digital exclusion is related to difficulty in finding lonely older adults. This suggests that other methods of connecting older adults to programs are needed. The most common solution has been the involvement of medical personnel in identifying lonely patients, sending them onward to social connectors via social prescribing (Sandset, Jentoft and Haldar, in press). Several sources also propose enlisting community members with public-facing jobs, such as hairdressers, cab drivers and store clerks, to act as connectors (7, 53, 73). This is in part because leafleting and contact by mail has reportedly proven ineffective (7). However, another solution involves what might be called “loneliness surveillance technologies”.

Loneliness surveillance technology encompasses a range of technologies including data sharing among organizations, as well as loneliness heat maps produced by charitable organizations (63, 68) or the public via apps. One app, Care View, allows neighbors to identify others who may be lonely, reporting them via a “government-backed” satellite equipped app. A press release for the UK Space Agency announcing a launch of the app in Leeds explains the basic premise:

People out in a community can tap the app when they spot signs that a householder may be struggling, like rubbish dumped in the garden or curtains that always remain shut. Through satellite technology, this “App Tap” generates a heat spot on a web-based map of the city, and if there are multiple “App Taps”, this creates heat-maps pinpointing streets and homes where people might welcome assistance of some kind (74).

Service workers, such as postal carriers, who are trained on how to reach out to lonely people, then contact said individuals. There is also some evidence in this press release that hard to find lonely people are so because of their socio-economic situation. In the release, Jon Hindley of Leeds City Council, remarks that “Care View has allowed us a window into the sometimes lonely and isolated world of vulnerable citizens within our poorest neighbourhoods” (74). This indicates certain areas are more likely to be targeted than others.

This project, however, raises ethical questions about the right to privacy and, moreover, the right to enjoy being alone. Perhaps being contacted on these grounds might be a stigmatizing, not to mention embarrassing experience. These apps are nevertheless pitched uncritically, without concern for the privacy or autonomy of older adults. The corpus also includes surveillance technologies like fall sensors, GPS tracking for people with dementia and cameras that allow older adults to live at home longer (see problematization two) in the range of technologies to diminish loneliness.

Problem representation four can be said to relate to the three previous representations in several ways. Those not digitally connected can be difficult to reach in a digitized society. Inadequate social care systems may overlook those who do not actively seek help, leading to unmet needs. Lonely older people, it is argued, are more likely to go unnoticed where public services are lacking, and infrastructure prevents frail older adults from leaving their homes. Additionally, family at a distance are less likely to realize their older family member is lonely and thus recommend appropriate social activities.

## Discussion

The argument thus far has been that four dominant problem representations can be discerned in the corpus, framing loneliness as a problem of non-use of technology, inadequate social provisions, geographic spread, and challenges of detection. As highlighted in the analysis, all four problematizations are interrelated. The next section offers reflection upon some possible repercussions of the dominant representations in relation to the existing literature. The primary focus lies in a critique of the dominant discourses, with the aim of fostering further discussion on how loneliness is constructed and its relation to technology moving forward. Following this discussion, possible alternatives are presented, some of which are found in the broader corpus.

Not only problems are brought into being through policy, but objects and political subjects as well. This is important because, as Davey and Glasgow argue, “assumptions about human nature frequently underpin policy proposals” and they shape “ways for people to be that can be harmful and limiting” (50). In the following, I therefore examine, both how loneliness is problematized and how older citizens and technology are constituted in the corpus.

### Some thoughts and challenges to the dominant problematizations

The various movements toward loneliness reduction in policymaking are presented as a politics of care. Documents in the corpus offer multiple calls to action and intervention, all practices of care, while older adults are primarily positioned discursively as recipients of that care. Care is also cause for concern. These policies reflect deep concernment with the public health consequences of loneliness and future economic costs that may be incurred upon the state by an aging population, alongside anxieties about societal change in the here-and-now.

These discourses reveal the emergence of loneliness as part of a broader sociotechnical imaginary (75). According to policymakers, digital developments have been beneficial–we have become connected to people around the world. Simultaneously, this development is said to come at the cost of face-to-face interactions as we progressively shop and obtain other services online. As families live at greater distances, neighborhood demographics radically change and available public spaces vanish, older people, it is claimed, grow increasingly alienated and isolated. Indeed, as several documents argue, we are possibly less connected to our neighbors than ever before. One remedy commonly offered in the loneliness policies is digital skills training.

Throughout the corpus, older adults are constituted as passive and lonely non-users of technology, indeed, victims of the digital age. Within this context, digital skills training is construed as a project of care and encourages the creation of care technologies. Paraphrasing de la Bellacasa, one can make oneself concerned about loneliness in society, but “to care” more strongly directs toward a notion of material doing (76). This problematization opens for forms of material doing, via the materiality of technologies. Teaching older adults to use the internet is also said to enable them to “care for” or “tend to” their social networks. So, this ethic of care through technology is understood to go both ways.

The belief that older adults are slow to the uptake of technology is both prevalent and pernicious (36, 77). Stereotypes about aging can become internalized, to detrimental effect (34). Successfully acquiring digital skills in older age has been tied to self-efficacy beliefs (29), however, those who internalize ageist stereotypes tend to have less self-efficacy regarding their ability to use technology (33). This potentially sets them up for failure. Lower engagement with technology may compound negative age-related beliefs about one's overall competence, whereas higher levels of use correlate with positive beliefs about aging and personal competence (37). Feasibly, if one fears being seen as incompetent due to the stereotype, one may be less likely to engage in digital skills training.

Corpus documents suggest skills training should ideally be performed by young volunteers. One might ask why older adults, such as retired IT experts, are not considered for this instruction. Policymakers clearly envision linking young people and older adults through digital skills training to stimulate inter-generational contact. However, they may not make the best instructors (27, 28). Researchers propose younger generations may take technological knowledge they possess for granted, not realizing older cohorts lack this knowledge. Older digital skills teachers better understand this disparity (29). One can also imagine seeing adults in their same age cohort as competent users could dismantle stereotypes, reduce stereotype threat, and boost self-efficacy. The reliance on young people as instructors could be reconsidered in this way. However, this becomes difficult to envision when older adults are universally constructed as non-users.

While some sources acknowledge that older adults use the internet by proxy via relatives, families are not positioned as ideal instructors in the corpus. The preferred approach is to bring in outsiders, rather than troubling the family. This diverges from other UK policies, which position the family as unpaid caregivers in other contexts (47). Some researchers have called the help families offer a double-edged sword. Family members can act as enablers, providing devices and offering tech support. However, they can also be impatient and cultivate feelings of incompetence (28, 77). It is worth noting that the provision, adaption and maintenance of said technologies can be one way families perform care (43). Research suggests a mix of formal digital skills training and family support is ideal (28, 77). The discourses in the corpus seem to exclude family involvement, omitting an important complement to formal skills instruction. If, as researchers suggest, families do have an important role to play in this project, formal skills training may fail when that care is discouraged or unavailable (28).

The choice to not be online is seldom presented as acceptable. More often, those who have made this choice are represented as unruly citizens who must be convinced of the value of digital connections. This reflects persisting normative beliefs that being online is always more desirable than not being online (78), along with an emerging normative belief that not being online is necessarily a catalyst for loneliness. The discourses also perpetuate assumptions that older adults do not already appreciate technology, or that they require help to understand why they should. These assumptions and normative beliefs impact the solutions that can be imagined. Only occasionally is it suggested that provisions must remain in place to maintain non-digital sources of information and service delivery for those who choose to remain offline (64). Research suggests that continuation of non-digital options benefit more demographics than just older adults (79), for example residents of rural areas (80).

Research also shows that some older adults do place value upon utilizing the internet for social support, using it to maintain current relationships, rather than to form new ones (24, 81, 82). This suggests skills training to mitigate loneliness among those who lack adequate social support networks may not be helpful. Interestingly, telephone contact, which is more familiar to the oldest adults and reported to mitigate loneliness by research participants (34), rarely appears in the discourse. Video calls and social media are instead emphasized as spaces where one can mingle intergenerationally and keep up with family.

Technologies created for older people often work from an “assistive, age-defined and deficit-focused” approach, while research suggests older people want to utilize the same technologies everyone else does (40). Video games, developed as sources of leisure for younger populations, could entice older adults to learn digital skills. Instead, game development takes on a utilitarian aspect when it comes to older adults (38). This seems to signal that developers do not consider leisure for the sake of leisure as important to the wellbeing of older people. It also may reflect discourses from grant initiatives that developers and researchers must mirror to successfully receive funding (38, 83).

Furthermore, older adults can at times be innovators when it comes to the uptake of technology (36). It is reasonable to believe coming generations of older adults will be increasingly more technologically adept. The question then becomes: what form(s) will the “problem” of loneliness among older adults take when all homes are “smart homes” and digital exclusion no longer functions as a feasible cause?

While technology in loneliness policy is pitched as a road toward independence, autonomy, and increased social interaction, this discourse is also is driven by the need to look ahead toward “cost-effective care”. This development cannot be examined without considering that social work with older people has been in decline for years. The shift is supported by active aging policies that simultaneously work against the needs of older people who do not fit the active aging discourse, further excluding them from society (22, 45). The push toward living at home as long as possible persists in UK policy, despite research that shows the home in older age can be experienced negatively, forming its own barriers to societal participation and potentially increasing loneliness (41). An interesting counter to the discourse is raised in the Welsh loneliness consultation. Here, a claim is made that the movement to have people live at home longer results in more people living alone vs. in group living arrangements where social activities could be easier to facilitate (67). However, homes for older adults can also bear stigma as symbols of what one does not desire in old age (48, 84). The ageing in place discourse (41) may perpetuate this stigma. Negative stereotypes about aging can also drive loneliness in older adults through withdrawal due to fear of threats to their identity (34).

The most common subject position afforded older adults in loneliness policy is one which constructs them as passive and helpless, perhaps a nuisance when they request help. It's easy to see how discourses like these might contribute to a phenomenon previous researchers have uncovered where older adults are discouraged from seeking assistance because they fear seeming incompetent and needy in a society which so clearly values independence (34). The UK has a long history of taking an active aging stance in other policy (41, 45, 47), however, in loneliness policy, “the passive victim” discourse Weicht (49) describes is far stronger. Weicht illuminates the problematic aspects of this ideal type, arguing that it strips older people of their role as actors, constructing them as a passive group things are “done-to” rather than as active participants in their own lives (49). Despite the presence of the active aging discourse in loneliness policy, in this context, older people are largely a monolith of passive subjects who must be helped toward becoming independent, active, non-lonely citizens. Only once they have reached this point do older adults regain some degree of agency in these politicized discourses.

Strangely, the care older people contribute to society, for example, through childcare and volunteering, is silent. Older people are recipients of interventions, not contributors; they need and take but do not give back. Lloyd et al. argue discursive constructions of older adults as dependent are harmful because they fail to acknowledge these continued contributions and oversimplify dependency issues (45). Although the value of volunteer work in relieving loneliness is emphasized throughout the corpus, little mention of the fact that pensioners comprise much of the volunteer sector is made. As with many discourses on care, one group (older adults), is constructed as “needy and dependent while ‘independent’ others attend to their ‘needs’”(85). The pro-surveillance and stigmatizing undertones of several of the assistive and loneliness surveillance technologies described in the corpus perhaps go unproblematized because older adults are constructed as passive non-actors.

Neven and Peine argue that because of its moral underpinnings, it is difficult to argue against the aging and innovation discourse. They claim it often serves to legitimate spending decisions, but may also lead to an uncritical acceptance of questionable technologies and waste (42). The same may be said of loneliness itself. Loneliness policy and the interventions they contain garner support across parties because of their moral underpinnings. Merged with the discourse of aging and innovation, it becomes even harder to argue against tech presented as a benevolent hero in the fight against loneliness in older adults.

### Are there discursive alternatives?

Loneliness prevention policies are presented as a call to come together and form a kinder society. Their neoliberal visions of a future where less reliance falls on the state, indeed on each other, then seem paradoxical. To offer a challenge to the dominant discourses presented, let us consider an alternative:

### Problematization representation 5: loneliness is a problem, not only of austerity, but neoliberalism itself

Communications, care, smart home, and surveillance technologies are appealing for the visions of connections, care cost reduction and independent living they invoke. As shown with the example from the Local Government Association, technology for loneliness is advantageous for those tasked with service delivery because it permits older adults to get by with less expensive forms of social care.

Simultaneously, recent trends in digitalization are problematized in loneliness policy. Several sources in the corpus raise concerns that, in addition to other problems that arise from digital exclusion, the grocery clerk replaced by online shopping may be the only face-to-face contact an older person has that day. One might reasonably ask how meaningful the contact is, and if it truly alleviates loneliness. Instead, dominant problematizations foster solutions that increase digital skills in older people and task civil society with providing that solution.

Constructing loneliness as a problem of digitalization and digital exclusion may direct attention (and funding) away from other problematizations. One such problematization in the corpus is one which understands the phenomenon as a problem of cuts to social programs that enable more meaningful contact than that provided by clerks and postal workers. Because dominant problematizations place the focus on digitalization, solutions like reversing budget cuts to reinstate social programs and public infrastructure to prior levels are deemphasized. The problem could even deter the digital integration of older people proposed in loneliness policy. For example, the digitally excluded often rely on public computers at libraries to access basic services. In 2019, funding for library computers with internet access was cut, in what Labour MP Tom Watson called a “digital exclusion double-whammy” (86). As I have shown, the problematization of loneliness as inadequate social care and public services is clearly present in the dominant discourse. How this problem is remedied is limited by other aspects of the discourse.

The 2007 financial crisis and austerity measures introduced by the Conservative-Liberal Democrat coalition government in its aftermath are credited with creating the biggest decline in welfare programs since the creation of the British welfare state (87). This continues a long-standing trend, as public social programs in the UK have been steadily eroded since the era of Thatcherism (88). This process continued through the transfer of responsibility for social programs to civil society in the coalition government's “Big Society” agenda (87, 89). McGimpsey states that these agendas operate as “an extension of neoliberal privatizing tendencies” while “‘austerity’ operates discursively in conjunction with ‘civil society’ as a means of maintaining cohesive internal relations of (a form of) the market state”(88). In fact, most loneliness strategies take a Big Society approach to the issue, relying on the third sector, business and communities to provide the interventions (90).

To illustrate this point, take one solution described in several documents: allocation of small development grants to the British tech sector. This makes the call for digital loneliness interventions lucrative for business. However, this effectively privatizes public funds by allowing the companies to maintain intellectual property rights for government-funded product development [see (91) for one example]. It also all but guarantees a market for said product.

Funding for social programs and public infrastructure improvements *is* allotted in loneliness policy, but the diverse corpus utilized in this study was able to illuminate challenges to these funding interventions. The challenges, largely found in debate excerpts from Labour party members, identify a fundamental gap in the funding allocated and the amount required to reinstate public service losses incurred under austerity policies. Although social care provision is criticized across parties as being in a dire state, mention of the political decisions that created the situation is scarce. Testimony from charitable organizations, like those found in the Welsh Government's loneliness inquiries (8, 67), attest to the detrimental precarity that comes with short-term grants such as those awarded following the release of the strategies.

Although the structural conditions that contribute to loneliness, like the closure of public spaces and a reduction in bus services is given attention in loneliness policy, the austerity measures that led to their elimination loom large but are rarely mentioned. These cuts have decimated communities and their public infrastructure, especially those which were already vulnerable. Shaw says, “Austerity can ruin the *there* of our *being-there*. By damaging the built environment, austerity gnaws at the human condition …” [(92) italics in original] leading to a state of alienation, isolation and uprootedness which Hannah Arendt believed to lie at the core of loneliness (93). Indeed, experiences of alienation from one's neighborhood are sometimes represented as an aspect of loneliness in policy, however, they are constructed as tied to demographic shifts and fear of crime, rather than previous policy decisions.

Several examples from this corpus cite modern living patterns as having effects on loneliness in older people. These include increases in the number of people living alone and families living at a distance. Some claims makers in the corpus, like the example cited from Lord Haselhurst, acknowledge that social and housing policies have contributed to the breakup of traditional living patterns. His concern is that tax laws dissuade families from living together or even nearby (64). One example is the “bedroom tax”, imposed to reduce housing benefits to those living in housing deemed “too large” for their needs. The policy was intended to force those with extra rooms (sometimes because a family member, such as a child had moved out) to move to smaller housing, freeing up space for larger families (94). This forced some to leave communities where they were socially rooted (95). While the policy only applied to working-aged people, older adults may have inadvertently been impacted if family members were forced to relocate. For those impacted directly, one study found that even when they did not move, participants' family relationships were impacted, as the extra burden meant some could no longer afford to travel to visit family or participate in social life (96). If loneliness is constructed as a problem of flawed policies, rather than one requiring digital contact, the possibility of repairing such flawed policy becomes apparent.

Discourses from the NHS have drawn criticism for their unquestioned assumption that living alone is an indicator of loneliness among older people (15). Most interesting, then, is the thought that digital connectedness and living independently, possible alone, are so adamantly believed to be the opposite of relying on and living in the company others in loneliness policy and political discourse. In addition to the cost-reduction benefits, positives like the peace of mind and independence telecare objects like fall detectors might bring older adults and their families are emphasized. Yet, little problematized is that such devices make invisible other aspects of care that simply cannot be provided by machines. Additionally, these technologies may further reduce the amount of face-to-face contact some older adults have. In the words of Mol, Moser and Pols: “[Care] may involve putting a hand on an arm at just the right moment, or jointly drinking hot chocolate while chatting about nothing in particular”(97). The move toward independence through technology may not be positioning older adults to receive the type of care they most desire and need. There is, after all, a difference between care that sustains and care that enriches life. However, cuts to social care have shortened human home care visits, eliminating the possibility for meaningful interactions (95).

Increased independence and decreased reliance on others, in this case supported by technology, is often uncritically accepted as a way out of loneliness. The idea that digital connectedness and living independently, possibly alone, are adamantly believed to be the opposite of relying on and living in the company others appears contradictory. Could a growing distance from and lack of reliance upon each other not play a role in the problem of loneliness in older adults? Policy that simultaneously laments a lack of connection between its citizens yet prizes independence and self-reliance above interdependency could be construed as part of the problem. One could ask if converting non-users to users of technology remains a viable solution when problematized as such.

An issue in the corpus related to problematization three is the prioritization of work over the social. This is unsurprising considering how neoliberal society produces subject positions whose worth is based worth upon their productivity (98). This focus on productivity does more than cause people to prioritize work over other important aspects of their life. It also leads to the demonization of dependency (98). This trend of positioning benefits claimants as a burden on the system extends back to the days of Thatcher. Under austerity, stigmatizing discourses and punitive policies intensified (95, 98–100). This stigmatization can cause those in need to feel deep shame when they utilize services, be it via public-funded benefit programs or charitable organizations (95, 98). As mentioned in debates within the corpus, older adults underclaim pension credits and thus miss out on other benefits to which they are entitled (64, 70). Goll and colleagues found participants who struggled with loneliness failed to claim benefits or accept help from charitable organizations because they did not wish to be perceived as a burden (34). We might ask ourselves if loneliness among older adults could in part be re-conceptualized as stemming from neoliberal norms that represent only productive citizens as worthy, while vilifying those in need. Changing this discourse only becomes a solution when loneliness is conceptualized in this way.

The strategies have been criticized for depoliticizing the loneliness issue through the omission of austerity and for their plans to mobilize communities, individuals, and civil society against loneliness in a climate of continued government spending cuts (95, 101). The problematization of loneliness as the result of austerity has not only been raised by Labour, but by interest groups (95), religious groups (102), academics (103–105) and the media (101, 106–110).

In an interview with former loneliness minister Tracey Crouch, she states, “Loneliness didn’t start in 2010. This is an issue that has been around in society for a very long time.” While she admits that funding issues may have impacted public services that facilitate social connection, she urges the public that her plan for taking on loneliness is about moving forward, not looking back (108). However, an ahistorical view of loneliness limits the discourse. Naturalizing loneliness as a timeless social problem precludes seeing it as shaped by past political decisions. This protects the parties that advocated for funding cuts by making causes diffuse, while maintaining constructs of austerity as an inexorable necessity.

Foucault asserted that understanding historical contexts and problematizations plays an important role in “diagnosing” the present. Through “[tracing] the struggles, displacements and processes of repurposing out of which contemporary practices emerged” we can understand the “historical conditions of existence upon which present-day practices depend” (111). A Foucauldian approach to discourse affords a remembering that cultivates a challenge to dominant ideologies (112). A look back permits the unearthing of alternatives that were championed, but fell to the wayside (113). Looking to the past and recontextualizing problematizations can reveal alternatives that challenge what today seems natural, even inevitable. In Bacchi's words, studying problematizations “politicizes taken-for-granted ‘truths’” (114).

Austerity measures were deemed necessary because government chose to bail out banks that caused the 2007 crash, plunging the UK into debt. Fearing the prospects of such debt on the economy, it made the public shoulder the costs, possibly for decades (87). Rather than place blame where due, austerity was sold to the public as a problem of the prior government's waste and overspending on the welfare state. It was legitimized as a necessary turn toward “frugality, self-sufficiency and fiscal prudence” (115). Its advocates also promised fairness, in that it would spare the most vulnerable (100). However, this turned out not to be the case.

Other solutions were proposed, solutions which problematized the country's economic straits in very different ways, but these other propositions were largely ignored (100). Conservative party members argue that austerity was a necessary evil, and the opposition parties seem to have forgotten that it may not have been. This is not to suggest that problematizing loneliness as related to economic decisions is the only explanatory device, but it does function as an alternative, one that enables us to see very different solutions.

One limitation of this study is that it covers a set timeframe of 2017–2021. A genealogical approach reaching further back in time may have identified other problematizations. Nevertheless, studying a broadly encompassing array of document types rather than extending the corpus chronologically enhanced the analysis and served the same purpose of unearthing alternative discourses. Future research might also explore how discourses evolved following the Covid-19 pandemic. It is possible that including documents without technology could have produced other results. However, the intended focus of this study was specifically how discourses of loneliness, technology and aging intersect. Given that all strategy documents located mention technology in some way, it's likely that dominant policy problematizations were represented.

“Refracting” the loneliness discourse through technology (58) in conjunction with Bacchi's WPR, helps to bring different problematizations to light. One illuminated alternative functions as an umbrella, covering many of the dominant problematizations presented. Technology plays multiple roles in loneliness policy. It is positioned as a driver, but when it comes to older people, it is also represented as a cure. Technology may be a favorable substitute for social care service expansion and enable further reduced spending on this population. However, if we reframe the problem of loneliness as one of austerity and neoliberalism, policymakers may have to ask themselves if this solution merely resembles a band-aid on the cracks of a disintegrating dam.

## Data Availability

The raw data supporting the conclusions of this article will be made available by the authors, without undue reservation.

## Footnotes

^1^One could ask if transportation and sidewalk infrastructure are not themselves kinds of technologies, however, in this article, the understanding of technology represented in the documents themselves is that which is drawn upon.

## References

[1] Storvik AG. Lanserte ensomhetsstrategi. [Released loneliness strategy]. Dagens Medisin [Internet]. (2019). Available at: https://www.dagensmedisin.no/politikk-og-okonomi/lanserte-ensomhetsstrategi/425735 (Accessed June 13, 2023).

[2] Ryall J. Japan: “Minister of loneliness” tackles mental health crisis. Deusche Welle [Internet]. (2021). Available at: https://www.dw.com/en/japan-minister-of-loneliness-tackles-mental-health-crisis/a-57311880 (Accessed June 13, 2023).

[3] Steenhard C. Opsamling på debat: Sådan skal Danmarks nationale ensomhedsstrategi se ud. [Togetherness on debate: This is how Denmark’s naitonal loneliness society will look]. Altinget Social. (2022) Available at: https://www.altinget.dk/social/artikel/opsamling-paa-debat-saadan-skal-danmarks-nationale-ensomhedsstrategi-se-ud (Accessed June 13, 2023).

[4] Dillinger K. Surgeon general lays out framework to tackle loneliness and “mend the social fabric of our nation.” CNN. (2023). Available at: https://edition.cnn.com/2023/05/02/health/murthy-loneliness-isolation/index.html (Accessed June 13, 2023).

[5] Tackling Loneliness annual report March 2023: the fourth year [Internet]. Department for Digital, Culture, Media & Sports (2023). Available at: https://www.gov.uk/government/publications/loneliness-annual-report-the-fourth-year/tackling-loneliness-annual-report-march-2023-the-fourth-year (Accessed May 30, 2023).

[6] Valtorta N Hanratty B. Loneliness, social isolation and health: “what’s the problem represented to be” in the United Kingdom? J Epidemiol Community Health. (2016) 70(Suppl. 1):A1–119. 10.1136/jech-2016-208064.135

[7] Tackling Loneliness in Merton. Merton City Council (2017). Available at: https://democracy.merton.gov.uk/documents/s18996/Tackling%20Loneliness%20in%20Merton.pdf (Accessed October 20, 2021).

[8] National Assembly for Wales. Inquiry into loneliness and isolation. National Assembly for Wales, Health, Social Care and Sport Committee (2017). Available at: https://business.senedd.wales/mgIssueHistoryHome.aspx?IId=16359 (Accessed October 20, 2020).

[9] Jopling K Aiden H. Loneliness and social isolation in the London Borough of Hounslow. (2017). Available at: https://democraticservices.hounslow.gov.uk/documents/s132656/Social%20Isolation%20-%20Appendix%201%20Loneliness%20and%20social%20isolation%20in%20the%20London%20Borough%20of%20Hounslow.pdf (Accessed October 20, 2020).

[10] Bacchi C. Problematizations in health policy: Questioning how “problems” are constituted in politics. SAGE Open (2016). p. 1–16. 10.1177/2158244016653986

[11] Bacchi C. Introducing the “what’s the problem represented to be?” approach. In: Bletsas A Beasley C, editors. Engaging with carol Bacchi. Adelaide, Australia: University of Adelaide Press (2012). p. 21–4.

[12] Mead R. What Britain’s “Minister of Loneliness” Says About Brexit and the Legacy of Jo Cox. The New Yorker. (2018). Available at: https://www.newyorker.com/culture/cultural-comment/britain-minister-of-loneliness-brexit-jo-cox (Accessed December 15, 2020).

[13] Jentoft EE Haldar M. Panacea or poison? Exploring the paradoxical problematizations of loneliness, technology and youth in Norwegian and UK policymaking. Int J Sociol Soc Policy. (2023). 10.1108/IJSSP-11-2022-0292

[14] Victor C Scambler S Bond J. The social world of older people: understanding loneliness and social isolation in later life. In: Walker A, editor. Understanding loneliness and social isolation in later life. Berkshire: McGraw-Hill Education (2009). p. 262.

[15] Hagan R. Loneliness, older people and a proposed social work response. J Soc Work. (2021) 21(5):1084–104. 10.1177/1468017320927630

[16] Sullivan MP Victor CR. Old and lonely: the loneliness narrative, moral regulation and the Media. Innov Aging. (2018) 2(S1):839. 10.1093/geroni/igy023.3126

[17] Ågren A. What are we talking about? Constructions of loneliness among older people in the Swedish news-press. J Aging Stud. (2017) 41:18–27. 10.1016/j.jaging.2017.03.002 28610751

[18] Victor C Mansfield L Kay T Daykin N Lane J Duffy LG An overview of reviews: the effectiveness of interventions to address loneliness at all stages of the life-course. What Works Centre for Wellbeing (2018). Available from: https://whatworkswellbeing.org/wp-content/uploads/2020/01/Full-report-Tackling-loneliness-Oct-2018_0151580300.pdf (Accessed November 19, 2020).

[19] Schirmer W Michailakis D. Loneliness among older people as a social problem: the perspectives of medicine, religion and economy. Ageing Soc. (2016) 36:1559–79. 10.1017/S0144686X15000999

[20] Ozawa-de Silva C Parsons M. Toward an anthropology of loneliness. Transcult Psychiatry. (2020) 57(5):613–22. 10.1177/1363461520961627 33076789

[21] Basu D. Ah, Look at all the lonely people….. will social psychiatry please stand up for ministering to loneliness? World Soc Psychiatry. (2021) 3(1):1–6. 10.4103/wsp.wsp_14_21

[22] Hagan R Manktelow R Taylor BJ Mallet J. Reducing loneliness amongst older people: a systematic search and narrative review. Aging Ment Health. (2014) 18(6):683–93. 10.1080/13607863.2013.875122 24437736

[23] Fakoya OA McCorry NK Donnelly M. Loneliness and social isolation interventions for older adults: a scoping review of reviews. BMC Public Health. (2020) 20(129). 10.1186/s12889-020-8251-6 32054474 PMC7020371

[24] Newman L Stoner C Spector A. Social networking sites and the experience of older adult users: a systematic review. Ageing Soc. (2021) 41:377–402. 10.1017/S0144686X19001144

[25] Campaign to End Loneliness. The psychology of loneliness: Why it matters and what we can do [Internet]. (2020). Available at: https://www.campaigntoendloneliness.org/wp-content/uploads/Psychology_of_Loneliness_FINAL_REPORT.pdf (Accessed September 02, 2020).

[26] Dickinson A Gregor P. Computer use has no demonstrated impact on the well-being of older adults. Int J Hum Comput Stud. (2006) 64:744–53. 10.1016/j.ijhcs.2006.03.001

[27] Holm IM Fagerlund AJ. Sosial digital kontakt [Social digital contact]. Nasjonalt senter for e-helseforskning; 2017. Available at: https://ehealthresearch.no/files/documents/Rapporter/NSE-rapport_2018-02_Sosial_digital_kontakt.pdf (Accessed September 02, 2020).

[28] Geertz N Schirmer W Vercruyssen A Glorieux I. Bridging the “instruction gap”: how ICT instructors help older adults with the acquisition of digital skills. Int J Lifelong Learn. (2023) 42(2):195–207. 10.1080/02601370.2023.2174197

[29] Schirmer W Geertz N Vercruyssen A Glorieux I, Digital Ageing Consortium. Digital skills training for older people: the importance of the “lifeworld.”. Arch Gerontol Geriatr. (2022) 101:104695. 10.1016/j.archger.2022.104695 35364451

[30] Doh M Schmidt LI Herbolsheimer F Jokisch M Wahl H. Patterns of ICT use among “senior technology experts”: the role of demographic variables, subjective beliefs and attitudes. In: Zhou J Salvendy G, editors. Human aspects of IT for the aged population design for aging ITAP 2015. Cham, Switzerland: Springer (2015). (Lecture Notes in Computer Science; vol. 9193). p. 177–88.

[31] Nef T Ganea RL Müri RM Mosimann UP. Social networking sites and older users—a systematic review. Int Psychogeriatr. (2013) 25(7):1041–53. 10.1017/S1041610213000355 23552297

[32] Olsson T Viscovi D. Who actually becomes a silver surfer? Prerequisites for digital inclusion. Javnost Public J Eur Inst Commun Cult. (2020) 27(3):230–46. 10.1080/13183222.2020.1794403

[33] Rosell J Vergés A. The impact of ageism on the E-leisure of older people in Chile. In: Gao Q Zhou J, editors. Human aspects of IT for the aged population technology design and acceptance. Cham: Springer (2021). p. 213–27. (Lecture Notes in Computer Science). Available at: https://link.springer.com/book/10.1007/978-3-030-78108-8

[34] Goll JC Charlesworth G Scior K Stott J. Barriers to social participation among lonely older adults: the influence of social fears and identity. PLoS One. (2015) 10(2):e0116664. 10.1371/journal.pone.0116664 25706933 PMC4338142

[35] Neves BB Waycott J Maddox A. When technologies are not enough: the challenges of digital interventions to address loneliness in later life. Sociol Res Online. (2023) 28(1):150–70. 10.1177/13607804211029298

[36] Loos E Peine A Fernandéz-Ardèvol M. Older people as early adopters and their unexpected and innovative use of new technologies: deviating from technology companies’ scripts. In: Gao Q Zhou J, editors. Human aspects of IT for the aged population technology design and acceptance. Cham: Springer (2021). p. 156–67. (HCII 2021: International Conference on Human-Computer Interaction; vol. 12786). Available at: https://link.springer.com/book/10.1007/978-3-030-78108-8

[37] Köttl H Tatzer VC Ayalon L. COVID-19 and Everyday ICT use: the discursive construction of old age in German media. The Gerontologist. (2022) 62(3):413–24. 10.1093/geront/gnab126 34436557 PMC8499783

[38] Iversen SM. Play and productivity: the constitution of ageing adults in research on digital games. Games Cult. (2016) 11(1–2):7–27. 10.1177/1555412014557541

[39] Lilja M Bergh A Johansson L Nygård L. Attitudes toward rehabilitation needs and support from assistive technology and the social environment among elderly people with disability. Occup Ther Int. (2003) 10(1):75–93. 10.1002/oti.178 12830320

[40] Light A Leong TW Robertson T. Ageing well with CSCW. In: Boulus RN Ellingsen G Bratteteig T Aanestad M Bjørn P, editors. ECSCW 2015: Proceedings of the 14th European Conference on Computer Supported Cooperative Work; 19–23 September 2015; Oslo, Norway. Berlin: Springer Cham (2015). p. 295–304.

[41] Sixsmith A Sixsmith J. Ageing in place in the United Kingdom. Ageing Int. (2008) 32(3):219–35. 10.1007/s12126-008-9019-y

[42] Neven L Peine A. From triple win to triple sin: how a problematic future discourse is shaping the way people age with technology. Societies. (2017) 7(26):377–402. 10.3390/soc7030026

[43] Greenhalgh T Wherton J Sugarhood P Hinder S Procter R Stones R. What matters to older people with assisted living needs? A phenomenological analysis of the use and non-use of telehealth and telecare. Soc Sci Med. (2013) 93:86–94. 10.1016/j.socscimed.2013.05.036 23906125

[44] Gilleard C Higgs P. Aging without agency: theorizing the fourth age. Aging Ment Health. (2010) 14(2):121–8. 10.1080/13607860903228762 20336545

[45] Lloyd L Tanner D Milne A Ray M Richards S Sullivan MP Look after yourself: active ageing, individual responsibility and the decline of social work with older people in the UK. Eur J Soc Work. (2014) 17(3):322–35. 10.1080/13691457.2013.829805

[46] Greenhalgh T Procter R Wherton J Sugarhood P Shaw S. The organising vision for telehealth and telecare: discourse analysis. BMJ Open. (2012) 2:e001574. 10.1136/bmjopen-2012-001574 22815469 PMC3401833

[47] Christensen K Pilling D. User participation policies in Norway and England—the case of older people and social care. J Soc Policy. (2019) 48(1):43–61. 10.1017/S0047279418000272

[48] Weicht B. Embracing dependency: rethinking (in)dependence in the discourse of care. Sociol Rev. (2011) 58(s2):205–24. 10.1111/j.1467-954X.2011.01970.x

[49] Weicht B. The making of “the elderly”: constructing the subject of care. J Aging Stud. (2013) 27(2):188–97. 10.1016/j.jaging.2013.03.001 23561284

[50] Davey J Glasgow K. Positive aging—a critical analysis. Policy Q. (2006) 2(4):21–7. 10.26686/pq.v2i4.4209

[51] LeGreco M Tracy SJ. Discourse tracing as qualitative practice. Qual Inq. (2009) 15(9):1516–43. 10.1177/1077800409343064

[52] Bacchi C Goodwin S. Poststructural policy analysis. New York: Palgrave Macmillan (2016).

[53] Department for Digital, Culture, Media & Sport. A connected society. A strategy for tackling loneliness - laying the foundations for change [Internet]. (2018). Available at: https://assets.publishing.service.gov.uk/government/uploads/system/uploads/attachment_data/file/750909/6.4882_DCMS_Loneliness_Strategy_web_Update.pdf (Accessed September 02, 2020).

[54] Jo Cox Loneliness. Combatting loneliness one conversation at a time. A call to action [Internet]. (2017). Available at: https://d3n8a8pro7vhmx.cloudfront.net/jcf/pages/164/attachments/original/1620919309/rb_dec17_jocox_commission_finalreport.pdf?1620919309 (Accessed September 02, 2020).

[55] The Jo Cox Foundation. The Jo Cox Loneliness Commission [Internet]. (2019). Available at: https://www.jocoxfoundation.org/loneliness_commission (Accessed June 18, 2023).

[56] Foucault M. The order of discourse. In: Young R, editors. Untying the text: a post-structualist reader. Boston: Routledge (1981). p. 51–77.

[57] Bacchi C. Policy as discourse: what does it mean? Where does it get US? Discourse Stud Cult Polit Educ. (2000) 21(1):45–57. 10.1080/01596300050005493

[58] Johnson E. Refracting spectrums of discourse. In: Refracting through technologies: Bodies, medical technologies and norms. Oxon, UK: Routledge (2020). p. 7–24.

[59] Scottish Government. A connected Scotland: our strategy for tackling social isolation and loneliness and building stronger social connections. [Internet]. Scottish Government. (2018). Available at: https://www.gov.scot/publications/connected-scotland-strategy-tackling-social-isolation-loneliness-building-stronger-social-connections/ (Accessed October 19, 2021).

[60] Bellis A. Tackling Loneliness. House of Commons Library (2019). Report No.: 8414.

[61] Hansard House of Commons. Loneliness and Local Communities [Internet]. Hansard. (2017). Available at: https://hansard.parliament.uk/Commons/2017-11-15/debates/D2106C26-6821-445C-8666-4F8D3445FFDA/LonelinessAndLocalCommunities (Accessed April 23, 2021).

[62] Welsh Government. Connected Communities. A strategy for tackling loneliness and social isolation and building stronger social connections [Internet]. Welsh Government. (2020). Available at: https://www.gov.wales/sites/default/files/publications/2020-02/connected-communities-strategy-document.pdf (Accessed October 20, 2021).

[63] Southampton City Council. Combating Loneliness in Southampton Scrutiny Inquiry—Draft [Internet]. Southampton City Council. (2017). Available at: https://www.southampton.gov.uk/media/0jjjnako/combating-loneliness-in-southampton-draft_tcm63-393674.pdf (Accessed October 20, 2021).

[64] Hansard House of Lords. Older Persons: Provision of Public Services [Internet]. Hansard. (2019). Available at: https://hansard.parliament.uk/Lords/2019-06-13/debates/5CC40CFB-2045-4E74-858A-B6C2CF5F22EB/OlderPersonsProvisionOfPublicServices?highlight=loneliness&hash;contribution-4054D467-CE8D-42B4-AA14-CE61B3E7AD01 (Accessed April 26, 2021).

[65] South Ayrshire Health and Social Care Partnership, NHS Ayrshire and Arran, South Ayrshire Council. Social Isolation and Loneliness Strategy 2018–2027 [Internet]. (2018). Available at: https://hscp.south-ayrshire.gov.uk/media/2652/Social-Isolation-Strategy-2019-2027/pdf/Social_Isolation_and_Loneliness_Strategy_2018-2027_v1.pdf?m=637667031244370000 (Accessed October 20, 2021).

[66] Hansard House of Commons. Loneliness Strategy [Internet]. Hansard (2018). Available at: https://hansard.parliament.uk/commons/2018-10-15/debates/3C48CE18-4473-4698-92E2-CF010D786CDC/LonelinessStrategy (Accessed September 20, 2022).

[67] Consultation—summary of responses. Connected Communities. Tackling Loneliness and Social Isolation [Internet]. Welsh Government. (2019). Report No.: WG35902. Available at: https://www.gov.wales/sites/default/files/consultations/2019-04/summary-of-responses.pdf (Accessed October 20, 2020).

[68] Local Government Association. Combating loneliness: A guide for local authorities [Internet]. Local Government Association. (2018). Available at: https://www.local.gov.uk/sites/default/files/documents/combating-loneliness-guid-24e_march_2018.pdf (Accesssed October 19, 2020).

[69] Department for Digital, Culture, Media & Sports, Office for Civil Society, James M. “Smart homes” to help older and disabled people get digital skills and tackle loneliness in rural areas [Internet]. (2019). Available at: https://www.gov.uk/government/news/smart-homes-to-help-older-and-disabled-people-get-digital-skills-and-tackle-loneliness-in-rural-areas (Accessed January 14, 2021).

[70] Hansard House of Commons. Free TV Licences: Over-75s [Internet]. (2019). Available at: https://hansard.parliament.uk/Commons/2019-06-11/debates/C43F0B6F-CC9C-437F-879F-FED5FF929A1B/FreeTVLicencesOver-75S?highlight=loneliness&hash;contribution-A1BF1CF4-DD28-4097-B353-B6F06E44F832 (Accessed April 26, 2021).

[71] Hansard House of Commons. Loneliness: Winter 2020–21. Volume 683: debated on Thursday 4 November 2020 [Internet]. (2020). Available at: https://hansard.parliament.uk/Commons/2020-11-05/debates/0F79F192-9786-44DE-B711-F442A339C116/LonelinessWinter2020-21?highlight=loneliness%20winter%202020-21&hash;contribution-32B770E8-C895-4613-A522-C3C0016FB0A7 (Accessed April 23, 2021).

[72] North Somerset Council. Social Isolation and Loneliness Needs Assessment and Strategy. North Somerset Council. (2019). Available at: https://www.n-somerset.gov.uk/sites/default/files/2020-03/social%20isolation%20and%20loneliness%20strategy%20-%20September%202019.pdf (Accessed October 20, 2021).

[73] North Somerset Council. Social Isolation and Loneliness Needs Assessment and Strategy [Internet]. (2019). Available at: https://www.n-somerset.gov.uk/sites/default/files/2020-03/social%20isolation%20and%20loneliness%20strategy%20-%20September%202019.pdf (Accessed October 20, 2021).

[74] UK Space Agency. Satellite-powered app to spot loneliness in hotspots in UK cities [Internet]. (2021). Available at: https://www.gov.uk/government/news/satellite-powered-app-to-spot-loneliness-in-hotspots-in-uk-cities (Accessed April 21, 2021).

[75] Jasanoff S. Future imperfect: science, technology, and he imaginations of modernity. In: Jasanoff S Kim S, editors. Dreamscapes of modernity. Chicago: University of Chicago Press (2015). p. 1–33.

[76] de la Bellacasa MP. Matters of care in technoscience: assembling neglected things. Soc Stud Sci. (2011) 41(1):85–106. 10.1177/0306312710380301 21553641

[77] Centre for Ageing Better. The digital age: new approaches to supporting people in later life get online [Internet]. Centre for Ageing Better. (2018). Available at: https://ageing-better.org.uk/sites/default/files/2018-06/The-digital-age.pdf (Accessed January 12, 2022).

[78] Wyatt S. Non-users also matter: the construction of users and non-users of the Internet. In: Oudshoorn N Pinch T, editors. How users matter. Cambridge, MA: MIT Press (2003).

[79] Hodkinson S Turner A Essen C. Exploring the impacts and implications of a changing UK welfare state under digitalisation and austerity: the case of leeds [Internet]. University of Leeds. (2016). Available at: https://eprints.whiterose.ac.uk/101701/ (Accessed June 10, 2023).

[80] Gerli P Whalley J. The impact of Brexit on the digitalisation of rural areas in the UK. In: Attorp A Heron S McAreavy R, editors. Rural governance in the UK. Milton Park, Abingdon, Oxfordshire: Routledge (2022).

[81] Kharicha K Manthorpe J Chew-Graham CA Cattan M Goodman C Kirby-Barr M Managing loneliness: a qualitative study of older people’s views. Aging Ment Health. (2021) 25(7):1206–13. 10.1080/13607863.2020.1729337 32091237

[82] Quan-Haase A Mo GY Wellman B. Connected seniors: how older adults in East York exchange social support online and offline. Inf Commun Soc. (2017) 20(7):967–83. 10.1080/1369118X.2017.1305428

[83] Vines J Pritchard G Wright P Olivier P Brittain K. An age-old problem: examining the discourses of ageing in HCI and strategies for future research. ACM Trans Comput-Hum Interact. (2015) 22(1):1–27. 10.1145/2696867

[84] Gubrium JF Holstein JA. The nursing home as a discursive anchor for the ageing body. Ageing Soc. (1999) 19(5):519–38. 10.1017/S0144686X99007448

[85] Beasley C Bacchi C. Envisaging a new politics for an ethical future. Beyond trust, care and generosity—towards an ethic of “social flesh”. Fem Theory. (2007) 8(3):279–98. 10.1177/1464700107082366

[86] Youle E. Exclusive: 4,000 Public Computers Slashed From Libraries And Jobcentres Under “Austerity Cuts.” Huffington Post UK [Internet]. (2019). Available at: https://www.huffingtonpost.co.uk/entry/public-computers-cut-from-libraries-jobcentres_uk_5c8a9e0ce4b0d7f6b0f0da14 (Accessed June 13, 2023).

[87] Ferguson I Lavalette M. Crisis, austerity and the future(s) of social work in the UK. Crit Radic Soc Work. (2013) 1(1):95–110. 10.1332/204986013X665992

[88] McGimpsey I. Late neoliberalism: delineating a policy regime. Crit Soc Policy. (2017) 37(1):64–84. 10.1177/0261018316653552

[89] Cabinet Office. Building the Big Society [Internet]. (2010). Available at: https://www.gov.uk/government/publications/building-the-big-society (Accessed January 14, 2021).

[90] Stenning A Hall SM. On the Frontline: Loneliness and the Politics of Austerity. Discover Society: On the Frontline [Internet]. (2018). Available at: https://archive.discoversociety.org/2018/11/06/on-the-frontline-loneliness-and-the-politics-of-austerity/ (Accessed October 19, 2020).

[91] Cabinet Office, Dowden O, CBE MP. Government uses innovative tech companies to tackle rural isolation and loneliness [Internet]. (2018). Available at: https://www.gov.uk/government/news/government-uses-innovative-tech-companies-to-tackle-rural-isolation-and-loneliness (Accessed January 14, 2021).

[92] Shaw IG. Worlding austerity: the spatial violence of poverty. EPD Soc Space. (2019) 37(6):971–89. 10.1177/0263775819857102

[93] Gaffney J. Another origin of totalitarianism: arendt on the loneliness of liberal citizens. J Br Soc Phenomenol. (2016) 47(1):1–17. 10.1080/00071773.2015.1097405

[94] Gibb K. The multiple policy failures of the UK bedroom tax. Int J Hous Policy. (2015) 15(2):148–66. 10.1080/14616718.2014.992681

[95] McGrath L Griffin V Mundy E Curno T Weerasinghe D Zlotowitz S. The psychological impact of austerity: a briefing paper. Educ Psychol Res Pract. (2016) 2(2):46–57. 10.15123/uel.885xw

[96] Mofatt S Lawson S Patterson P Holding E Dennison A Sowden S A qualitative study of the impact of the UK “bedroom tax.”. J Public Health (Bangkok). (2015) 38(2):197–205. 10.1093/pubmed/fdv031 PMC4894481 25774056

[97] Mol A Moser I Pols J. Care: putting practice into theory. In: Mols A Moser I Pols J, editors. Care in practice. Bielefeld, Germany: Transcript (2010). p. 7–25.

[98] Mills C. “Dead people don’t claim”: a psychopolitical autopsy of UK austerity suicides. Crit Soc Policy. (2018) 38(2):302–22. 10.1177/0261018317726263

[99] Stanley L. Legitimacy gaps, taxpayer conflict, and the politics of austerity in the UK. Br J Polit Int Relat. (2016) 18(2):389–406. 10.1177/1369148115615031

[100] Fairclough I. Evaluating policy as argument: the public debate over the first UK austerity budget. Crit Discourse Stud. (2016) 13(1):57–77. 10.1080/17405904.2015.1074595

[101] Grieg J. Loneliness is a political problem. Tribune [Internet]. (2022). Available at: https://tribunemag.co.uk/2022/11/loneliness-is-a-political-problem (Accessed June 10, 2023).

[102] Barclay D Hilhorst S. Holy alliances: church-secular partnerships for social good [Internet]. Demos. (2019). Available at: https://demos.co.uk/research/holy-alliances-church-secular-partnerships-for-social-good/ (Accessed December 11, 2020).

[103] Brownlee K Jenkins D. Improving the loneliness strategy [Internet]. Warwick Social Sciences Policy Briefing. (2019). Available at: https://warwick.ac.uk/fac/soc/impact/policybriefings/1_improving_the_loneliness_strategy.pdf (Accessed October 19, 2020).

[104] Bridger O Evans R. Tackling loneliness and social isolation in reading, England. University of Reading Participation Lab, University of Reading, Reading UK [Internet]. 2019. Available at: https://research.reading.ac.uk/participation-lab/wp-content/uploads/sites/131/Unorganized/Bridger-and-Evans-2019-Tackling-Loneliness-in-Reading-report.pdf (Accessed October 19, 2020).

[105] Dobson J. Green spaces help combat loneliness - but they demand investment. The Conversation [Internet]. (2018). Available at: https://theconversation.com/green-spaces-help-combat-loneliness-but-they-demand-investment-105260 (Accessed October 20, 2020).

[106] Butler P. The “despair” and “loneliness” of austerity Britain. The Guardian [Internet]. (2012) Available at: https://www.theguardian.com/society/2012/jul/17/despair-loneliness-austerity-britain (Accessed October 20, 2020).

[107] Yeginsu C. “This is all we can afford”: shrinking lives in the english countryside. The New York Times [Internet]. (2019). Available at: https://www.nytimes.com/2019/05/13/world/europe/cumbria-uk-austerity-cuts.html (Accessed October 20, 2020).

[108] Roberton J. Loneliness didn’t start in 2010—but Tory austerity has had impact, minister tells ITV News. ITV News [Internet]. (2018). Available at: https://www.itv.com/news/2018-10-15/loneliness-didnt-start-in-2010-but-tory-austerity-has-had-impact-minister-tells-itv-news (Accessed June 10, 2023).

[109] Safety net cuts, not just modern life, are making people lonely, critics say [Internet]. KCUR, NPR. (2019). Available at: https://www.kcur.org/health/2019-06-19/safety-net-cuts-not-just-modern-life-are-making-people-lonely-critics-say (Accessed October 19, 2020).

[110] Is austerity making Britain lonely? [Internet]. Sky News. (2018). Available at: https://news.sky.com/video/is-austerity-making-britain-lonely-11236774 (Accessed June 10, 2023).

[111] Garland D. What is a ‘“history of the present”? On Foucault’s genealogies and their critical preconditions. Punishment Soc. (2014) 16(4):365–84. 10.1177/1462474514541711

[112] Medina J. Toward a Foucaultian epistemology of resistance: counter-memory, epistemic friction and guerilla pluralism. Foucault Stud. (2011) 12:9–35. 10.22439/fs.v0i12.3335

[113] Hook D. Genealogy, discourse, “effective history”: Foucault and the work ofcritique. Qual Res Psychol. (2005) 2:3–31. 10.1191/1478088705qp025oa

[114] Bacchi C. Why study problematizations? Making politics visible. Open J Polit Sci. (2012) 2(1):1–8. 10.4236/ojps.2012.21001

[115] MacLeavy J. A “new politics” of austerity, workfare and gender? The UK coalition government’s welfare reform proposals. Camb J Reg Econ Soc. (2011) 4:355–67. 10.1093/cjres/rsr023

